# Short-Term Effects of Tillage Practices on Soil Organic Carbon Turnover Assessed by *δ* ^13^C Abundance in Particle-Size Fractions of Black Soils from Northeast China

**DOI:** 10.1155/2014/514183

**Published:** 2014-08-05

**Authors:** Aizhen Liang, Shenglong Chen, Xiaoping Zhang, Xuewen Chen

**Affiliations:** ^1^Northeast Institute of Geography and Agroecology, Chinese Academy of Sciences, Changchun 130102, China; ^2^University of Chinese Academy of Sciences, Beijing 100049, China

## Abstract

The combination of isotope trace technique and SOC fractionation allows a better understanding of SOC dynamics. A five-year tillage experiment consisting of no-tillage (NT) and mouldboard plough (MP) was used to study the changes in particle-size SOC fractions and corresponding *δ* ^13^C natural abundance to assess SOC turnover in the 0–20 cm layer of black soils under tillage practices. Compared to the initial level, total SOC tended to be stratified but showed a slight increase in the entire plough layer under short-term NT. MP had no significant impacts on SOC at any depth. Because of significant increases in coarse particulate organic carbon (POC) and decreases in fine POC, total POC did not remarkably decrease under NT and MP. A distinct increase in silt plus clay OC occurred in NT plots, but not in MP plots. However, the *δ* ^13^C abundances of both coarse and fine POC increased, while those of silt plus clay OC remained almost the same under NT. The C derived from C_3_ plants was mainly associated with fine particles and much less with coarse particles. These results suggested that short-term NT and MP preferentially enhanced the turnover of POC, which was considerably faster than that of silt plus clay OC.

## 1. Introduction

In the past few years physical fractionation has played an important role in studying soil organic carbon (SOC) pool and its turnover under different land uses and management systems [1]. The stable C isotope trace technique is increasingly used to study the dynamics of SOC and the influences of agricultural managements on SOC turnover [2, 3]. However, SOC dynamics is so complex that it is hard to tell how SOC pool responds to changes in land uses and soil managements if only total SOC is measured. Thus, a combination of stable C isotope trace technique and SOC fractionation can be used to explore the mechanism of C dynamics and quantify the accumulation of newly added SOC in different SOC fractions [4]. Piao et al. [5] found that in forest soils, the *δ* ^13^C values of SOC in light fractions were significantly lower than in heavy fractions, indicating that crop residues were first incorporated into the light fractions. The SOC in the light fraction or coarse particles was labile and sensitive to changes of soil managements [5, 6]. However, Magid et al. [7] found that labile SOC fractions were distributed in all-sized or all-density particles. Yonekura et al. [8] also found that when forest was converted to grassland, all soil organic matter fractions in the surface layer exhibited fast turnover based on natural abundance of *δ* ^13^C.

Long-term intensive agricultural management could greatly reduce SOC level [9]. Also, it is well documented that conservation tillage could enhance C protection, increase SOC levels [10–13], and convert agricultural soils from C sources to C sinks, thereby removing significant amounts of CO_2_ from the atmosphere [14]. However, the effect of conservation tillage on SOC storage is sometimes contradictory [15, 16], especially for short-term (≤10 years) effect of conservation tillage. It varies with soil conditions, such as soil texture, climate, and biomass return, as well as management itself, and is difficult to be generalized [10, 17]. Hence, the studies on short-term impacts of tillage systems on SOC have to be verified in different soil conditions.

Northeast China is an important agriculture region, mainly due to the production of soybean and maize. Black soil (Typic Hapludoll, US Soil Taxonomy) dominates this region and mainly occurs in Liaoning, Jilin, and Heilongjiang provinces. Since 1940s and 1950s, large-scale cultivation and improper management have resulted in a significant decline of soil fertility, and present SOC content is less than one-half of the initial content before cultivation [18]. Hence, it is essential to find out an appropriate tillage system which can enhance the SOC level, while maintaining or improving local crop production. The objectives of this study were to analyze the dynamics of particle-size SOC fractions and to explore SOC turnover using *δ* ^13^C natural abundances under short-term tillage practices in black soils of Northeast China.

## 2. Materials and Methods

### 2.1. Study Site

The tillage study was started in the fall of 2011 at the Experimental Station (44° 12′ N, 125° 33′ E) of Northeast Institute of Geography and Agroecology, Chinese Academy of Sciences, in Dehui, Jilin Province, China. The study site is located in the North Temperate Zone with a continental monsoon climate. Mean annual temperature is 4.4°C, and mean annual precipitation is 520.3 mm, with more than 70% occurring from June to August [18]. The soil was classified as a black soil, following the Chinese Soil Classification System, equivalent to a Typic Hapludoll in the Soil Taxonomy. This soil was clay loam textured, with an average of 36% clay, 24% silt, and 40% sand. Before the experiment started, the soil bulk density was 1.24–1.38 g cm^−3^. The total SOC and nitrogen (N) contents were 15.5 g kg^−1^ and 1.31 g kg^−1^, respectively. The slope of the experimental plots is less than 1°. The land had been used for continuous corn production under conventional tillage for many years (≥20 years) prior to 2001 [19].

### 2.2. Tillage Experiment

A tillage experiment, consisting of two treatments, mouldboard plough (MP) and no tillage (NT), was established in a randomized complete block design with four replicates. Each tillage plot was split into two subplots (5.2 m × 20 m) which were under corn-soybean rotation with both crops present each year. The MP included fall mouldboard ploughing (about 20 cm deep) after corn harvest, spring disking (7.5 to 10 cm deep), and field cultivation. The NT soils had no soil disturbance except for crop planting using a KINZE-3000 NT planter (Williamsburg, Iowa). All the crop residues were returned to the soil surface. Each year, 100 kg ha^−1^ N was applied to corn as starter fertilizer and 50 kg ha^−1^ N as top dressing at the V-6 growth stage, respectively. In addition, 45.5 kg ha^−1^ P and 78 kg ha^−1^ K were also added to corn as starter fertilizers. For soybean, all fertilizers were applied as starter fertilizer, including 40 kg ha^−1^ N, 60 kg ha^−1^ P, and 80 kg ha^−1^ K. The starter fertilizers for all plots were applied concurrently during the planting phase.

### 2.3. Soil Sampling and Measurements

Composite soil samples (7 subsamples per plot) were collected down to a depth of 20 cm after harvest (corn phase) in 2001 and 2006. The samples were taken using a hand auger (2.64 cm diameter) which allowed separation of each soil core into three segments (0–5, 5–10, and 10–20 cm) without soil compaction. Soil samples were gently broken and air-dried. Visibly identifiable crop residues were manually removed for SOC and SOC fractions measurements.

The silt plus clay (<20 *μ*m) SOC was measured as described by Liang et al. [20]. 25 g dry soil was placed in a 250 mL beaker and 125 mL distilled water was added. The soil suspension was mixed and allowed to settle at room temperature overnight. Then, the suspension was dispersed for 10 min with an ultrasonic probe (JY92 and 24 kHz, Xinzhi, Ningbo) with an energy input of 480 J mL^−1^. The dispersed soil suspension was transferred to a 1 L glass cylinder, and the cylinder was capped and shaken end over end to thoroughly homogenize the soil water suspension. Silt plus clay fraction and clay fraction were collected by siphoning the suspension based on Stokes' law. The collected solid fractions were oven-dried at 60°C and then ground for SOC analysis. Silt OC content was calculated by the silt plus clay OC minus clay OC.

Soil particulate organic carbon (POC, 53–2000 *μ*m) was measured according to the method of Carter et al. [21]. 20 g soil sample passing through 2 mm sieve was put into a plastic vial and 100 mL 5 g L^−1^ sodium hexametaphosphate solution was added. The sample was shaken for 2 h for dispersion. The dispersed suspension was passed through a set of 250 and 53 *μ*m sieves from top to bottom. Distilled water was used to rinse soil particles on the sieves. Soil fractions retained on the sieves were collected and oven-dried at 60°C and then ground for SOC analysis. The POC in the >250 *μ*m size was defined as coarse POC, and POC in the 53–250 *μ*m size was fine POC.

All soil samples were free of carbonate, and hence SOC content was assumed to be equal to total C content. The total SOC and SOC fractions were determined using a FlashEA1112 elemental analyzer (ThermoFinnigan, Italy). Soil subsamples of 20–40 mg and plant samples (including natural plant and corn residues) were ground and passed through a 100-mesh sieve and then were used to measure the *δ* ^13^C abundances of SOC by an isotope ration mass spectrograph (MAT252, Finnigan, USA).

### 2.4. Data Analysis

The proportion of C derived from corn was calculated using [22]

(1) $$\begin{array}{c} f=\frac{\left(\delta -\delta_{0}\right)}{\left(\delta_{1}-\delta_{0}\right)}, \end{array}$$

where *f* stands for the proportion of corn-derived C in the sample,  *δ* for the measured *δ* ^13^C values in corn fields, *δ*_0_ for the *δ* ^13^C of the corresponding sample from corn field as C_3_ reference soil, and *δ*_1_ for the *δ* ^13^C values of corn plant (about −12‰).

Turnover time of SOC was calculated according to [22]

(2) $$\begin{array}{c} T=\frac{1}{k}=\frac{-\left(t-t_{0}\right)}{\ln\left(C_{t}/C_{t0}\right)}, \end{array}$$

where *k* stands for the rate constant of the first-order decay, *t* for the year of sampling (2006), *t*_0_ for the year of vegetation change, *C*_*t*_ for the remaining proportion of C_3_–C at the time of sampling (%), and *C*_*t*0_ for the proportion of C_3_–C at *t*_0_ (100%).

Least significant difference (LSD) between means was calculated to examine the effects of tillage practices on SOC in each fraction. The procedure was performed using SAS 9.1 (SAS Institute, Cary, NC, USA). Statistical significance was determined at *P* < 0.05.

## 3. Results and Discussion

### 3.1. Total and Particle-Size Soil Organic Carbon under NT and MP

Five-year NT resulted in no substantial changes (1.2%) in total SOC in top 0–20 cm soil compared to the initial level. However, there were significant increases (9.9%) in total SOC at 0–5 cm depth (*P* < 0.05), despite a lack of noticeable differences at 5–20 cm depths after 5-year NT. MP had no significant effects on total SOC contents in the entire plough layer (*P* > 0.05). This difference was likely not a result from the difference in biomass inputs [23], but rather it resulted from retention of carbon from crop residue on the soil surface of the NT plots whereas it was incorporated into the soil in the MP plots.

**Figure 1:**
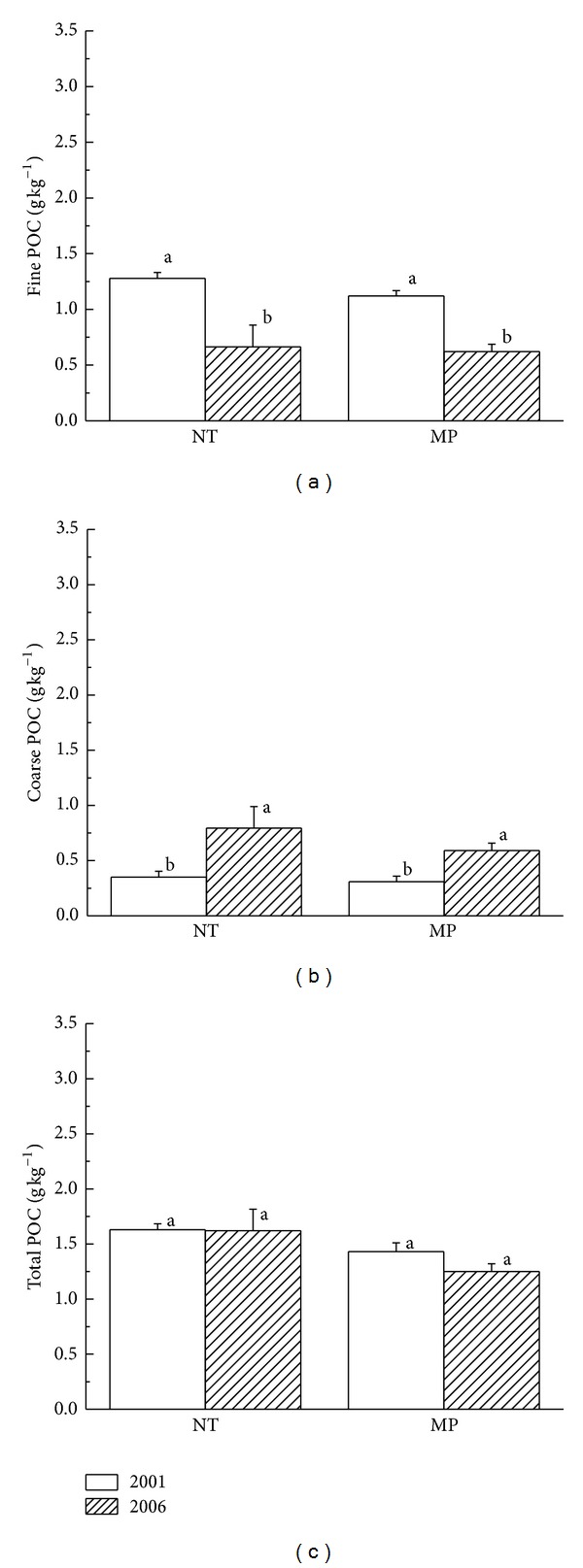
Particulate organic carbon (POC) in 0–20 cm layer of black soil under no tillage (NT) and mouldboard plough (MP). Bars indicate standard error. Different lowercase letters above the column indicate significant differences at *P* = 0.05.

Over five years, NT and MP significantly affected both fine and coarse POC (*P* < 0.05, Figures 1(a) and 1(b)). Due to significant and opposite influences of tillage treatments on fine and coarse POC, total POC contents had no distinct changes (Figure 1(c)). There were significant increases in coarse POC under NT and MP (*P* < 0.05) which could be attributed to the annual input of organic materials. The NT decreased fine POC, which was different from the result of Pikul et al. [24]. We considered that our experimental study was conducted for a short period (only five years) which was not sufficient for crop residue decomposition and subsequent C association with finer soil particles. Collectively, these results suggested that SOC in coarse particle was more sensitive to the conversion of tillage systems.

**Figure 2:**
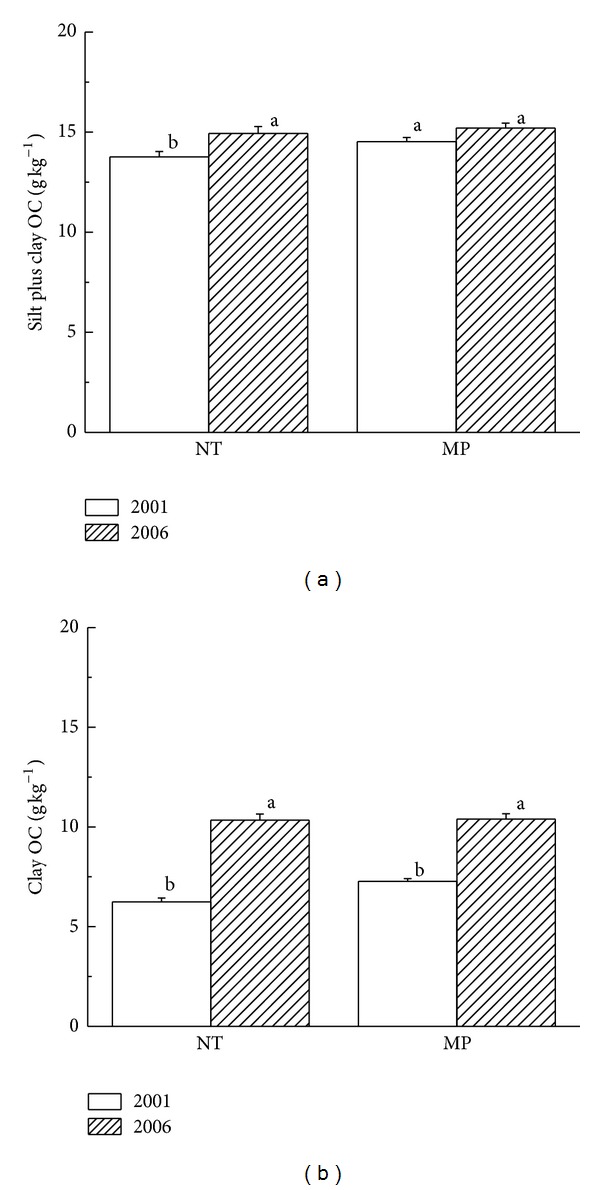
Soil organic carbon in <20 *µ*m particles of the 0–20 cm layer under tillage practices.

Under NT and MP treatments, silt plus clay OC and clay OC in the plough layers both showed an increasing trend over the five-year period (Figures 2(a) and 2(b)). Compared to data in 2001, the NT treatment significantly increased 7.9% and 39.7% SOC in these two size fractions (*P* < 0.05). These results indicated that short-term NT decreased silt OC, whereas clay OC played an important role in the total increase in silt plus clay OC. The reason was that the retention of crop residues above the soil surface and no disturbance under NT reduced the loss of silt plus clay OC. The MP only increased silt plus clay OC by 4.5% (*P* > 0.05). Similar to NT plots, there were significant increases in clay OC in the MP plots by 30.0% on average (*P* < 0.05). However, the concurrent decrease in silt OC and the increase in clay OC suggested that SOC in each fraction probably was translocated by splash erosion of rainfall or was transferred between fine and coarse particles [25]. The increase in silt plus clay OC under MP might be due to the breakdown of macroaggregates into microaggregates by tillage practice and incorporation of crop residues into soils which increased the input of organic materials. Also, undecomposed crop residues to some extent prevented soil from splash erosion. Hence, there were increases in silt plus clay OC in MP plots, but the increases were less than those in NT plots under greater protection of crop residues on the surface.

### 3.2. *δ* ^13^C Abundances in Organic Carbon Fractions under NT and MP

At 0–20 cm depths the *δ* ^13^C abundances (−19.9‰ and −21.1‰) of SOC in both fine and coarse fractions were lower than those of corn plants (−12.0‰) (Table 1). This was because the study site was conducted under corn-soybean rotation system; soybean residues (C_3_) were returned into soils in addition to corn residues (C_4_). Neither NT nor MP significantly affected the *δ* ^13^C values of SOC fractions (Table 1). Gregorich and Janzen [26] reported that the changes in *δ* ^13^C values of SOC with depths could approximately reflect the characters of SOC decomposition. In the present study, the *δ* ^13^C values of SOC in each size fraction decreased with depths under NT and MP treatments (Table 1), which could be related to the decreasing input of C_4_ plants from top to subsurface soils. Average *δ* ^13^C values of POC in the coarse and fine fractions in 0–20 cm layers were −20.3‰ and −19.4‰ in the NT plots, respectively, slightly greater than that of silt plus clay OC (−21.0‰). Similar results were found in MP plots.

Factors affecting the *δ* ^13^C values include plant (plant types and water use efficiency of plant), soil (total SOC, SOC fractions, soil depths, and soil moisture), landform, and fertilization [27]. In this study, soil was the primary factor affecting *δ* ^13^C values at the experimental site because of the same plant, landform, and fertilization. In NT plots higher soil moisture [28, 29] resulted in lower *δ* ^13^C values of SOC in each fraction than in MP plots. It was due to the return of crop residues each year. Certainly the inputs and decomposition of organic materials were different between the NT and MP plots. Without fall ploughing, crop residues returned to the field significantly increased SOC concentration at 0–5 cm depth under NT [19]. We therefore hypothesized that in our present study *δ* ^13^C values of SOC in each fraction under NT were lower than under MP. However, the experimental result was not exactly consistent with the above hypothesis. Neither NT nor MP practice significantly affected *δ* ^13^C values of SOC fractions, despite the existence of differences (Table 1). In 0–20 cm layers, the *δ* ^13^C values of coarse and fine POC were both slightly greater in NT plots (−20.3‰ and −19.4‰) than in MP plots (−20.5‰ and −19.5‰), whereas the *δ* ^13^C values of silt plus clay OC were almost the same under two tillage treatments. Above results might be due to other soil conditions. Walley et al. [30] reported that NT increased soil water supplement and water use efficiency of plants. The higher the water use efficiency is, the higher the *δ* ^13^C values are [31]. In our case, crops with higher abundances of *δ* ^13^C were returned and then increased soil *δ* ^13^C values. Thus, the *δ* ^13^C values of coarse and fine POC at 0–20 cm depth were greater under NT than under MP. Additionally, the interactions among relevant factors might affect the *δ* ^13^C values. Collectively, the above results suggested that short-term impacts of tillage practices on SOC were shown in the coarse fractions, with no substantial changes in fine particles.

### 3.3. Plant Sources of Soil Organic Carbon under NT and MP

In NT soils C_3_–C and C_4_–C concentrations in coarse POC fraction significantly decreased (*P* < 0.05), and the former was greater than the latter (Table 1). The C_3_–C and C_4_–C concentrations in fine POC showed the same variation trend, though the changes were not significant (*P* > 0.05). In MP soils, C_3_–C and C_4_–C concentrations of POC in the fine and coarse fractions had no noticeable decreases at two depths. These different effects were attributed to the enrichment of organic materials on soil surface under NT treatment in contrast to the incorporation of returned crop residues into soils and thus even distribution of organic materials inputs in plough layers under MP.

There were no significant differences in C_3_–C and C_4_–C concentrations in each SOC fraction between the NT and MP treatments (*P* > 0.05) (Table 1). The NT only increased C_3_–C and C_4_–C concentrations in coarse POC at 0–10 cm depth. In other SOC fractions, both C_3_–C and C_4_–C concentrations were lower in NT soils than in MP soils, which could be attributed to the stratification of SOC in soil profiles under NT. In addition, the result indicated that the sensitive response of SOC to tillage conversion could be shown in the coarse fraction. There were no significant differences in the proportions of C_3_–C and C_4_–C between the NT and MP treatments (*P* > 0.05, Table 1). Short-term NT practice thus could not significantly affect the C source of SOC fractions, even POC. Regardless of the tillage practices (NT and MP), C derived from C_3_ plants was mainly distributed in the fine-size fractions (Table 1). That is, the turnover of SOC was faster in the coarse-size fraction than in fine-size fraction, which is in agreement with studies of Yonekura et al. [8] and Yamashita et al. [22].

### 3.4. Turnover Rates of Soil Organic Carbon under NT and MP

**Figure 3:**
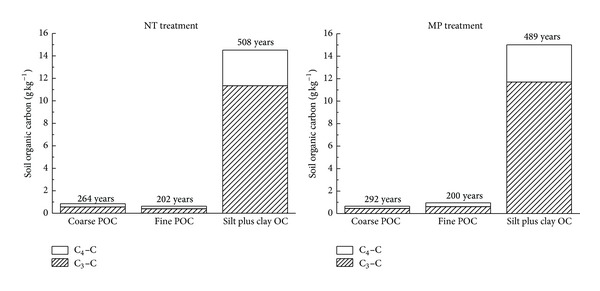
Concentrations of C_3_–C and C_4_–C in different fractions and the mean turnover time of C under tillage practices.

The silt plus clay OC represented not only the majority of the total SOC, but also the SOC fraction with the longest apparent turnover time under NT and MP practices (Figure 3). The turnover time of POC in the NT and MP soils was more than 200 years, far longer than the results previously reported by Cambardella and Elliott [32] that the turnover time of POC ranged from 5 to 20 years in cultivated grassland soils. The reason might be that after cultivation of virgin black soils, soybean (C_3_ crop) residues provided an extra source of organic matter input in addition to corn-derived C (C_4_ crop). It might also be due to a certain amount of black C in POC [33]. The mean turnover time indicated faster turnover of SOC in coarse fraction than that in fine fraction. We suggested that short-term NT did not significantly affect the turnover time of SOC. The turnover time of SOC was even longer in MP plots because of the incorporation of returned crop residues into soils.

## 4. Conclusions

Five years of no tillage tended to stratify total SOC concentration in the plough layer (0–20 cm) on the studied clay loam soil of Northeast China. Soil management using no tillage on this fine-textured and poor-drained black soil might not sequester more SOC than conventional tillage. No tillage had great impacts on fine and coarse POC, though the total POC did not significantly increase. Compared with mouldboard plough, no tillage remarkably increased silt plus clay OC in the plough layer. Both no tillage and mouldboard plough did not significantly affect *δ* ^13^C value in each SOC fraction, despite the occurrence of certain changes. The *δ* ^13^C values of coarse and fine POC under no tillage treatment were greater than those under mouldboard plough, but there were almost no changes in *δ* ^13^C values of silt plus clay OC between these two treatments. Thus, the short-term impact of no tillage was firstly shown in the coarse-size fractions (POC). The distribution of C_3_–C mainly in fine particles (silt plus clay) indicated that the turnover of SOC in coarse-size fraction was faster under tillage practices. Further study is needed to verify the long-term effects of tillage practices on SOC turnover and distribution in different size fractions.

## Figures and Tables

**Table 1:** *δ* ^13^C abundances, C_3_–C and C_4_–C fractions in the plough layer of Black soil under no tillage (NT) and mouldboard plough (MP) in 2006.

	Treatments	*δ* ^ 13^C (‰)	*f* (%)	1 − *f* (%)	C_4_–C (g kg^−1^)	C_3_–C (g kg^−1^)
0–10 cm						
Coarse POC	NT	−19.8	32.2	67.8	0.48	0.83
	MP	−20.2	32.1	67.9	0.27	0.58
Fine POC	NT	−19.3	40.8	59.2	0.32	0.47
	MP	−19.2	41.2	58.8	0.43	0.60
Silt plus clay OC	NT	−21.1	27.4	72.6	4.00	10.6
	MP	−21.1	27.2	72.8	4.10	11.0

10–20 cm
Coarse POC	NT	−20.8	25.3	74.7	0.10	0.29
	MP	−20.7	25.8	74.2	0.12	0.34
Fine POC	NT	−19.5	30.4	69.6	0.14	0.33
	MP	−19.8	27.3	72.7	0.26	0.63
Silt plus clay OC	NT	−21.1	16.1	83.9	2.34	12.1
	MP	−21.1	17.1	82.9	2.54	12.4
